# Concurrent Presentation of Euryblepharon and Moyamoya Syndrome in Costello Syndrome: A Rare Clinical Case

**DOI:** 10.7759/cureus.40808

**Published:** 2023-06-22

**Authors:** Asma M Alhazmi, Musab A Alsubaie, Reem R Alanazi

**Affiliations:** 1 Ophthalmology Department, King Fahd Hospital of the University/Imam Abdulrahman Bin Faisal University, Dammam, SAU

**Keywords:** facio-cutaneous-skeletal syndrome, hras, rasopathy, costello syndrome, moyamoya syndrome, euryblepharon

## Abstract

This case report provides a detailed examination of a rare co-occurrence of Costello syndrome, euryblepharon, and Moyamoya syndrome in a 14-year-old female. Costello syndrome, a rare genetic disorder characterized by developmental delays, distinctive facial characteristics, and a predisposition to certain malignancies, presents an array of ocular manifestations, including downward-slanting palpebral fissures. A significant similarity is noted with euryblepharon, a rare periocular anomaly marked by the downward slanting of the eyelids. Despite these striking resemblances, the association between euryblepharon and Costello syndrome is yet to be documented in the literature. Furthermore, the coexistence of Costello syndrome and Moyamoya syndrome, a cerebrovascular disorder, is exceedingly rare. This report provides an in-depth analysis of the patient's ocular and periocular manifestations, establishing a potential association of euryblepharon within the phenotypic spectrum of Costello syndrome and documenting the unusual co-occurrence with Moyamoya syndrome. These findings aim to augment our understanding of Costello syndrome's phenotypic variability and potential associations.

## Introduction

Costello syndrome, an uncommon genetic disorder with a prevalence estimated between one in 300,000 to one in 1.25 million, typically manifests with a spectrum of clinical attributes, such as developmental delays, unique facial characteristics, and an increased predisposition to particular malignancies [1]. Among these facial features, downward slanting of the palpebral fissure is commonly noted, complemented by a diverse array of ocular manifestations.

Euryblepharon, a rare periocular anomaly, is characterized by horizontal elongation and vertical shortening of the eyelids, along with an inferior displacement of the lateral canthal tendons. This leads to the characteristic downward-slanting appearance of the lateral palpebral fissure. This bears a striking resemblance to the palpebral fissure's downward slanting, a frequently described feature in Costello syndrome. Despite the notable similarity, an explicit association between euryblepharon and Costello syndrome remains to be documented in the existing literature [2].

Furthermore, the co-occurrence of Costello syndrome and moyamoya syndrome, a cerebrovascular pathology, is exceptionally rare, adding to the complexity and variability of the Costello syndrome phenotype [3].

This case report aims to elucidate an intriguing clinical picture of a 14-year-old female with Costello syndrome, potentially extending our understanding of the phenotypic variability and potential associations related to this syndrome.

## Case presentation

A 14-year-old female, known to have Costello syndrome, epilepsy, and moyamoya syndrome, was referred to our oculoplasty department. The patient's chief complaints were persistent tearing and foreign body sensation in both eyes, symptoms she had been experiencing for a long duration. Notably, she had undergone lacrimal probing six years prior at an external institution.

Upon examination, her corrected visual acuity was 20/50 OD and 20/125 OS, and her intraocular pressure was 19 mmHg OD and 21 mmHg OS. A refractive analysis identified myopic astigmatism -4.50 -1.75 x 10 OD and -6.00 -1.00 x 175 OS.

**Figure 1:**
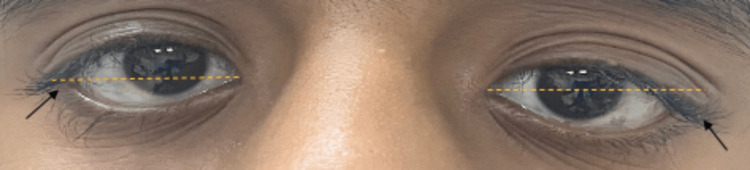
Note the flat nasal bridge and the horizontal elongation of the eyelids with an inferior displacement of the lateral canthal tendons and downward slanting (black arrows)

In terms of periocular examination, we observed malar hypoplasia, bilateral euryblepharon with inferior displacement of the lateral canthus relative to the medial canthus, as shown in Figure 1.

Additional findings included bilateral lower lid retraction, nasally located trichiasis OU, multiple bilateral eyelid folds, and 2-mm medially accentuated lagophthalmos. During our assessment of extraocular motility, we documented nystagmus, superior oblique palsy with inferior oblique overaction OU, and a -1 deficit in abduction OU. The Krimsky test indicated 30 prism dioptre esotropia OS and dissociated vertical deviation. Slit-lamp examination revealed superficial punctate keratopathy OU, and dilated fundus examination identified tilted discs OU.

A treatment regimen was initiated to manage amblyopia, utilizing part-time occlusion OD. The most recent follow-up disclosed corrected visual acuities of 20/50 OD and 20/40 OS. During the initial visits, epilation was performed to manage trichiasis. Electrolysis, though the definitive treatment, was not done due to the medial location of the misdirected lashes near the punctum and canalicular system.

The patient's previous medical assessments and a genetic study had been conducted earlier in life at a separate hospital. She had exhibited developmental delay, primarily cognitive and speech delay, in addition to dysmorphic coarse facial features and a notably large head circumference (53.5 cm). Neurological evaluation recorded mild hypotonia, preserved deep tendon reflexes, and normal gait. Her epilepsy presented as focal seizures, which were effectively managed with carbamazepine.

Whole exome sequencing revealed a heterozygous mutation of the HRAS gene, a class I pathogenic variant, confirming the diagnosis of Costello syndrome.

Furthermore, the patient's medical history included episodes of recurrent left-sided weakness exacerbated by crying. Brain magnetic resonance imaging illustrated narrowed supraclinoid segments of both internal carotid arteries and the presence of prominent perforators and collaterals around the expected sites of the middle cerebral artery and anterior cerebral artery, aligning with the characteristics of moyamoya syndrome.

## Discussion

Costello syndrome, first delineated by the New Zealand pediatrician Costello in the 1970s, is a distinct genetic disorder marked by pronounced failure-to-thrive, developmental delays, distinctive coarse facial characteristics, and a heightened predisposition to specific malignancies [4,5]. The causative etiological factor for Costello syndrome is established to be germline mutations in the HRAS proto-oncogene, a critical component in the regulation of cellular proliferation and differentiation [6].

Costello syndrome is recognized as part of the RASopathies, a group of syndromes caused by germline mutations in genes that are part of the RAS/MAPK pathway, which includes neurofibromatosis type 1, Noonan syndrome, and cardiofaciocutaneous syndrome, among others. These syndromes share many overlapping features, underscoring their common genetic etiology [7].

Costello syndrome presents with a diverse range of ophthalmic and periocular findings. Hypertelorism, a salient facial dysmorphology in this syndrome, is routinely observed [8]. The periocular findings are varied and often comprise prominent or edematous eyelids, epicanthal folds, and down-slanting palpebral fissures. Ophthalmic manifestations commonly include strabismus, nystagmus, and a range of refractive errors, from myopia to hypermetropia. Ptosis, lacrimal duct anomalies, and keratoconus have also been reported, expanding the clinical phenotype associated with Costello syndrome [8].

The diverse manifestations observed in our patient provide a tangible illustration of the extensive phenotypic spectrum associated with HRAS dysregulation. Furthermore, they underscore the importance of a comprehensive ophthalmic examination in the effective management of patients diagnosed with Costello syndrome.

Euryblepharon, an uncommon congenital palpebral anomaly, manifests as a simultaneous transverse elongation and vertical contraction of the eyelids. This presentation is typically accompanied by the inferior displacement of the lateral canthal tendon, ultimately resulting in the distinctive downward-slanting appearance of the lateral palpebral fissure. In most instances, euryblepharon presents in a bilateral and symmetrical manner, with a predilection for the lower eyelid [2].

Upon analyzing the body of literature regarding Costello syndrome, a marked prevalence of downward-slanting palpebral fissures is apparent in the reported cases. A comprehensive literature review conducted by Hennekam, involving 103 patients diagnosed with Costello syndrome, elucidated that 40 out of 47 patients demonstrated downward-slanting palpebral fissures [9]. This ocular feature, recurrently observed in Costello syndrome, mirrors the clinical presentation of euryblepharon. Despite this evident overlap in clinical presentation, the term 'euryblepharon' remains largely unreported in the documented studies on Costello syndrome.

Given the pronounced similarities in the clinical manifestation of these two conditions, we propose that euryblepharon should be recognized as a distinct, defining feature in the phenotypic spectrum of Costello syndrome.

Moyamoya disease, first described in Japan by Takeuchi and Shimizu in 1957, is a cerebrovascular disorder characterized by progressive stenosis of the internal carotid arteries and their branches within the circle of Willis [10]. This constriction is often accompanied by the development of a compensatory network of small, fragile vessels, a pathology that results in compromised cerebral blood flow and an increased risk of transient ischemic attacks, strokes, and cognitive decline [11]. While the etiology of moyamoya disease is largely idiopathic, familial occurrence and genetic associations have been noted, particularly in populations of Asian descent [12].

Moyamoya syndrome refers to similar cerebrovascular changes but is associated with a variety of known diseases or occurs following specific treatments. Conditions such as Down syndrome, neurofibromatosis type 1, and sickle cell disease, among others, can precipitate these vascular changes [11]. Furthermore, moyamoya syndrome can develop as a late complication following cranial irradiation therapy [13].

Interestingly, there has been an emerging body of literature highlighting a potential association between RASopathies and moyamoya syndrome. Certain RASopathies, particularly neurofibromatosis type 1, have been frequently observed in individuals diagnosed with moyamoya syndrome [14].

While the underlying molecular mechanisms linking RASopathies and moyamoya syndrome remain to be fully elucidated, this association suggests a potentially shared pathogenic pathway and underscores the importance of vigilant neurological assessment and monitoring in individuals with RASopathies.

Despite this, the co-existence of Costello and moyamoya syndromes is exceptionally rare, with the first such instance documented by Shiihara et al. [3]. Our patient stands as the second reported case in medical literature to simultaneously have Costello and moyamoya syndromes, thereby adding to our collective knowledge of this unusual pairing of conditions.

## Conclusions

In conclusion, our case report addresses unexplored aspects of this clinical condition, contributing to the existing knowledge in this area. Our primary objective is to establish a clear association between euryblepharon and Costello syndrome, an association that, to our knowledge, has yet to be explicitly outlined in previous reports despite conspicuous similarities in the clinical presentations of the two conditions.

Additionally, our report aims to provide a comprehensive description of our patient's ocular and periocular manifestations, thereby enhancing the existing descriptions of these symptoms within the context of Costello syndrome. Finally, we report an unusual co-occurrence of Costello and moyamoya syndromes within a single patient, a rare conjunction of conditions that provide further insights into the potential complexities of Costello syndrome's phenotypic spectrum.
